# The connecting brain in context: How adolescent plasticity supports learning and development

**DOI:** 10.1016/j.dcn.2024.101486

**Published:** 2024-11-28

**Authors:** Amanda E. Baker, Adriana Galván, Andrew J. Fuligni

**Affiliations:** University of California, Los Angeles, CA, USA

**Keywords:** Adolescence, Environment, Plasticity, Learning, Brain development

## Abstract

Puberty initiates significant neurobiological changes that amplify adolescents’ responsiveness to their environment, facilitating neural adaptation through processes like synaptic pruning, myelination, and neuronal reorganization. This heightened neuroplasticity, combined with their burgeoning social curiosity and appetite for risk, propels adolescents to explore diverse new environments and forge social bonds. Such exploration can accelerate experiential learning and the formation of social networks as adolescents prepare for adult independence. This review examines the complex interplay between adolescent neuroplasticity, environmental influences, and learning processes, synthesizing findings from recent studies that illustrate how factors such as social interactions, school environments, and neighborhood contexts influence both the transient activation and enduring organization of the developing brain. We advocate for incorporating social interaction into adolescent-tailored interventions, leveraging their social plasticity to optimize learning and development during this critical phase. Going forward, we discuss the importance of longitudinal studies that employ multimodal approaches to characterize the dynamic interactions between development and environment, highlighting recent advancements in quantifying environmental impacts in studies of developmental neuroscience. Ultimately, this paper provides an updated synopsis of adolescent neuroplasticity and the environment, underscoring the potential for environmental enrichment programs to support healthy brain development and resilience at this critical development stage.

## 1 Introduction

Adolescence is a critical stage of socio-emotional and cognitive development marked by profound changes in brain structure and function (Casey et al., 2008, Larsen and Luna, 2018). This period begins with the onset of puberty, when a surge of hormones acts as a catalyst for neural remodeling by initiating key processes such as synaptic pruning (trimming of unused neural connections), myelination (formation of protective sheaths that enhance neural transmission), and neuronal reorganization (restructuring of neural networks) (Juraska, 2024, Pfeifer and Allen, 2021, Sisk and Zehr, 2005). These neurobiological changes heighten neuroplasticity—the brain’s remarkable capacity to adapt in response to experience—thereby laying the foundation for learning and development (Galván, 2010, Larsen and Luna, 2018, Lin et al., 2020).

Rather than unfolding uniformly across the brain, puberty-related changes involve a series of intricate and interacting processes that are most pronounced within the neural networks relevant to adolescent behavior and development (Delevich et al., 2021, Juraska, 2024, Sydnor et al., 2023). For example, heightened activation of the mesolimbic dopamine system is thought to motivate adolescents to explore the world, take risks, and learn from experiences, stimulating their curiosity for acquiring new skills and knowledge (Galvan, 2010). Simultaneously, puberty-related changes in social brain systems can make adolescents more attuned to social cues (De Lorme et al., 2013, Pfeifer and Allen, 2021), supporting their ability and motivation to navigate complex social interactions and form meaningful social relationships (Bos and van den, 2013, Crone and Dahl, 2012, Sullivan et al., 2022). Through these experiences, adolescents can build the skills they need to navigate their increasingly complex world, make adaptive decisions, and pursue their long-term goals (Crone and Dahl, 2012, Dow-Edwards et al., 2019, Wilbrecht and Davidow, 2024).

Prolonged plasticity of the prefrontal cortex, a critical hub for executive function and behavioral regulation, likely plays an important role in helping adolescents adapt to changing contexts as connections within and between brain networks are streamlined (Delevich et al., 2021, Lin et al., 2020, Sydnor et al., 2023). However, neuroplasticity may render adolescents particularly vulnerable to environmental stressors, which can impact neural development and contribute to adolescent-onset mental health disorders such as anxiety and depression (Drzewiecki and Juraska, 2020, Merikangas et al., 2010). Thus, providing adolescents with supportive environments is crucial for promoting their well-being into adulthood.

The heightened neuroplasticity characteristic of adolescence presents a critical opportunity for intervention and prevention efforts aimed at fostering positive developmental outcomes (Cousijn et al., 2018, Crone and Dahl, 2012, Laube et al., 2020). To fully harness this potential, a nuanced understanding of how plasticity and experience interact to shape learning processes in the journey through puberty is essential. The field is at an exciting juncture, with emerging research methodologies and analytical tools providing new avenues to explore the complexities of adolescent neuroplasticity and its interaction with environmental factors. Building on foundational developmental research, recent studies have uncovered novel insights into the mechanisms underlying adolescent neuroplasticity, the timing of sensitive periods, and the impact of environmental contexts on adolescent development. This review aims to synthesize these findings, highlighting their implications for adolescent brain development and cognitive and socio-emotional outcomes, as well as discussing open areas of inquiry that warrant further exploration. By focusing on recent research (e.g., studies published in the past 5 years) with appropriate sample sizes, we seek to integrate new discoveries about neuroplasticity across diverse social, educational, and neighborhood environments.

This review emphasizes the need for continued investigation, advocating for future research to embrace multimodal, longitudinal designs and highlighting recent efforts to better characterize environmental effects on adolescent brain development. Ultimately, this work underscores the importance of tailored environmental enrichment interventions and policy strategies that align with the distinct neurodevelopmental trajectories of adolescents, thereby fostering positive outcomes that extend into adulthood.

## 2 Puberty, plasticity, and the potential for learning

Puberty is a pivotal developmental phase marked by intersecting hormonal, environmental, and social changes, creating a sensitive window for experiential learning and neural reorganization (Sisk, 2017). This section explores how gonadal hormones (e.g., estrogen and testosterone) directly affect neurodevelopment, and how their interactions with evolving social and environmental contexts collectively shape socio-cognitive development from adolescence into adulthood (Larsen and Luna, 2018).

### 2.1 Hormonal effects on neurodevelopment

Pubertal hormone surges exert both activational and organizational effects on brain development. Activational effects involve temporary changes in brain function that occur when hormones like estrogen and testosterone bind to receptors in key brain regions, including the ventral striatum (e.g., nucleus accumbens; NAcc), dorsal striatum (e.g., caudate and putamen), amygdala, hippocampus, and prefrontal cortex (PFC; Laube et al., 2020; Peper and Dahl, 2013). This hormone-receptor binding modulates neural activity and neurotransmitter dynamics within systems critical for reward, motivation, social cognition, and cognitive control, such as the mesolimbic dopamine system (Caldú and Dreher, 2009, Peters and Naneix, 2022). For instance, estrogen can enhance dopaminergic signaling by upregulating dopamine receptor expression and dendritic spine density in the NAcc, heightening neural reward sensitivity (Laube et al., 2020, Peper and Dahl, 2013, Schulz and Sisk, 2016, Zehr et al., 2006). While activational effects are transient (De Lorme et al., 2013), they can indirectly contribute to longer-term organizational changes by impacting how adolescents engage with their environments (Delevich and Wilbrecht, 2020).

Unlike temporary activational effects, organizational effects involve enduring structural and functional changes in the brain during critical periods like puberty (De Lorme et al., 2013, Schulz and Sisk, 2016, Sisk and Zehr, 2005). These changes include processes such as synaptic pruning, long-term potentiation, and alterations in neural connectivity that together contribute to the gradual consolidation and refinement of neural networks supporting complex socio-cognitive functions (De Lorme et al., 2013, Schulz and Sisk, 2016, Sisk and Zehr, 2005). Importantly, these organizational changes in the brain persist after pubertal hormone surges subside, highlighting the profound impact of pubertal hormone exposure during adolescence on neural maturation (De Lorme et al., 2013, Schulz and Sisk, 2016, Sisk and Zehr, 2005).

#### 2.1.1 Brain network reorganization

In early adolescence, regions across the cortex exhibit anatomical similarities (e.g., similar cortical thickness), suggesting a phase of structural convergence before the brain specializes into distinct networks (Vijayakumar et al., 2021). As adolescence progresses, higher-order association areas involved in critical thinking and behavioral control—such as the PFC—undergo significant structural maturation that enhances their connectivity, establishing their role as hubs for broader neural communication (Vijayakumar et al., 2021). Meanwhile, mesolimbic dopamine circuits continue reorganizing, with dopamine axons growing from the striatum to the PFC, strengthening prefrontal-subcortical neural pathways (Hoops and Flores, 2017, Reynolds and Flores, 2021).

Functional brain network reorganization during puberty involves increases in centrality (importance of network hub regions), segregation (formation of specialized subnetworks), efficiency (efficiency of network communication), and integration (communication between specialized regions), particularly in attention, task control, and social processing systems (Gracia-Tabuenca et al., 2021). Functional brain network development occurs along a sensorimotor-association axis, with lower-order sensorimotor areas (e.g., motor cortex) maturing earlier and integrating functionally with other regions, while higher-order association areas (e.g., PFC) continue maturing and functionally specializing throughout adolescence (Larsen et al., 2023, Luo et al., 2024, Sydnor et al., 2023).

#### 2.1.2 Neuroplasticity

Neuroplasticity in adolescence also follows a similar sensorimotor-association trajectory. Research examining intrinsic cortical activity fluctuations—which have been linked to neuroplasticity in animal models—suggests that sensorimotor plasticity declines from childhood to adolescence, while plasticity in association areas, especially the PFC, continues to increase until mid-adolescence (Sydnor et al., 2023). This extended prefrontal plasticity, combined with increasing independence and curiosity for exploring their surroundings, likely supports adolescents’ ability to process diverse socio-emotional inputs and adapt flexibly to new environmental demands, creating a critical window for developing complex socio-cognitive skills (Delevich et al., 2021, Drzewiecki and Juraska, 2020, Juraska, 2024, Larsen and Luna, 2018, Nelson and Guyer, 2011, Wilbrecht and Davidow, 2024). However, these dynamics can also amplify the impact of environmental stressors on brain function and behavior, emphasizing the importance of supportive and enriching environments during this sensitive developmental window (Drzewiecki and Juraska, 2020, Hoops and Flores, 2017, Sydnor et al., 2023).

#### 2.1.3 The hypothalamic–pituitary–adrenal (HPA) axis

The hypothalamic–pituitary–adrenal (HPA) axis, a key regulator of stress responses, undergoes extensive reorganization during puberty, amplifying adolescents’ reactivity to environmental stressors (Romeo, 2010). Prior to pubertal onset, rats exhibit elevated levels of stress-related hormones in the pituitary and adrenal glands, potentially contributing to heightened hormonal stress reactivity (Kann and Romeo, 2022). As puberty unfolds, stress-related hormones interact with influxes of gonadal hormones, leading to sex-dependent shifts in stress reactivity (Romeo, 2010, Romeo et al., 2016). These cycles are further intensified by adolescents’ exposure to stress, which modulates both stress-related and pubertal hormone levels (Romeo and Sciortino, 2021) with interactive downstream effects on dopamine transmission and dopamine-related behaviors (Sinclair et al., 2014). The reprogramming of the HPA axis during puberty and its effects on developing neural systems can make adolescents especially vulnerable to stress, with significant implications for their socio-emotional development into adulthood.

In summary, the activational and organizational effects of pubertal hormones create a dynamic and impressionable neurobiological landscape characterized by reward sensitivity, stress reactivity, and social motivation. Together, these transient and enduring changes make adolescence a critical period for brain and behavioral development, establishing the foundation for healthy adult functioning (Larsen and Luna, 2018).

### 2.2 Social plasticity

Adolescence is a critical period of social reorientation marked by heightened sensitivity to social stimuli and increased exposure to diverse social environments (Bos and van den, 2013, De Lorme et al., 2013, Dow-Edwards et al., 2019). Hormonal and environmental shifts during this time encourage the pursuit of social interactions, which are essential for the development of behavioral responses and complex social skills necessary for navigating adult social contexts (Dow-Edwards et al., 2019, Nelson and Guyer, 2011). As adolescents adapt their behavior based on social feedback, the neural circuits involved in social processing are refined, leading to lasting organizational changes in the structure and function of social brain networks (De Lorme et al., 2013). This heightened social sensitivity and enhanced capacity to learn from social information is referred to as “social plasticity” (Bos and van den, 2013, Dow-Edwards et al., 2019).

Social plasticity reflects how social experiences activate neuroplasticity in adolescence, facilitating learning by strengthening connections within and between neural and social networks. It operates through two primary mechanisms: experience-expectant plasticity, where the brain is primed for social input (e.g., heightened neural sensitivity to social cues), and experience-dependent plasticity, where social experiences sculpt brain networks (e.g., neural reorganization following specific social experiences; De Lorme et al., 2013; Galván, 2010). Social interactions can also activate meta-plastic processes, whereby experiences extend periods of developmental neuroplasticity, allowing for continued learning and neural flexibility (Frankenhuis and Walasek, 2020). By integrating social and cognitive processes, social plasticity can help adolescents pursue goals aligned with their social and educational environments, such as building social networks, participating in collaborative activities, and developing complex interpersonal skills.

#### 2.2.1 Insights from animal research

Research in non-human species offers valuable insights into the neurobiological mechanisms underlying social plasticity (Adret, 2022, Arnold, 1992, Bölting and Von Engelhardt, 2017, Nordeen and Nordeen, 1990). For example, in juvenile songbirds, vocal learning is associated with neurogenesis in the auditory system, illustrating social experience-dependent plasticity in adolescence (Arnold, 1992, Nordeen and Nordeen, 1990). In mice, adolescent social isolation leads to dendritic spine excess and hyperactivity in the PFC, interfering with flexible goal-seeking and reward pursuit and promoting habit-based behavior (Li et al., 2024). The cusp of puberty (post-weaning days 21–35 in mice) has been identified as a critical window during which social isolation alters prefrontal myelination and impairs social behavior—effects that persist despite later social reintroduction (Makinodan et al., 2012). Social isolation during this window further disrupts maturation of prefrontal interneurons that trigger social approach behaviors, leading to long-term social deficits (Bicks et al., 2020). Importantly, chemogenetic activation of these interneurons in adulthood partially mitigated these deficits, demonstrating the potential of targeted neural modulation to restore aspects of social plasticity (Bicks et al., 2020).

These findings suggest the pre/peri-pubertal window is a sensitive period for social learning during which social enrichment interventions may be particularly effective. However, more cross-species research is required to precisely map these neural circuits in humans and develop targeted interventions that combine neural circuit modulation with behavioral strategies to support adolescent social development.

#### 2.2.2 Social plasticity and puberty

Adolescent social development is shaped by puberty-dependent and puberty-independent factors. A meta-analysis of neuroimaging studies found that puberty consistently modulates neural responses during social information processing, suggesting that puberty is central to the social reorientation of adolescence in humans (Dai and Scherf, 2019). However, animal studies suggest that some social behaviors emerge before or independently of puberty. For example, in rats, prepubertal ovariectomy disrupts social recognition abilities, which cannot be reversed with later estradiol administration, suggesting that gonadal hormones play an essential role in organizing the neural circuits responsible for social recognition prior to pubertal onset (Yoest et al., 2023). In hamsters, neither altering pubertal timing nor prepubertal gonadectomy affected the age at which social play shifted to aggression; however, both manipulations increased play and reduced aggression, suggesting that while some social behaviors emerge independently of puberty, gonadal hormones modulate the intensity and expression of these behaviors to match developmentally appropriate levels (Paul et al., 2018). In songbirds, testosterone accelerates the consolidation of learned songs and crystallizes the ability to learn new songs, suggesting that gonadal hormones can facilitate and constrain social learning depending on developmental needs (Templeton et al., 2012). These findings underscore the complex interplay between pubertal hormonal changes and intrinsic developmental processes in shaping adolescent social behavior, emphasizing the need for further research to disentangle these mechanisms.

#### 2.2.3 The social plasticity hypothesis

The social plasticity hypothesis posits that while puberty-related increases in social sensitivity may initially elevate risky behaviors (e.g., substance use), changing social norms and continued brain maturation help mitigate these behaviors later in adolescence—explaining why many substance use disorders resolve without formalized intervention before adulthood (Cousijn et al., 2018). Simulations of adolescent decision-making support this hypothesis, indicating that adolescents learn quickly from social information, stabilizing their behavior and reducing exploratory actions over time (Ciranka and van den Bos, 2021). Beyond substance use, the social plasticity hypothesis offers a valuable framework for understanding how the social reorientation of adolescence fosters adaptive learning. Just as heat can help mold a substance, leveraging social plasticity may enhance programs designed to foster positive developmental trajectories in adolescence.

### 2.3 Mechanisms of experiential learning in adolescence

Puberty-related changes in mesolimbic dopamine dynamics and shifting environmental and social factors contribute to increases in reward sensitivity, exploration, and risk-taking in adolescence (Parr et al., 2024). These behaviors are thought to play an adaptive role in learning, enhancing neuroplasticity and promoting the development of cognitive flexibility from adolescence into adulthood (Parr et al., 2024).

#### 2.3.1 Reward sensitivity

Adolescents’ capacity for learning is closely tied to their ability to adjust behavior based on feedback—particularly rewards (McCormick and Telzer, 2017b, Palminteri et al., 2016). During adolescence, developmental increases in prefrontal and striatal reward sensitivity are correlated with improvements in flexible learning (McCormick and Telzer, 2017a), highlighting the role of developing mesolimbic circuitry in adolescents’ ability to engage with and adapt to uncertain environments (Hoops and Flores, 2017, Parr et al., 2024). These patterns are not limited to humans; for example, adolescent rats display improved performance on reversal-learning tasks due to their responsiveness to positive outcomes (Afshar et al., 2020). This reward responsivity during adolescence not only facilitates immediate learning but also predicts better performance in adulthood (Afshar et al., 2020), illustrating how reward-related processes in adolescence can help establish adaptive behaviors that persist later in life.

However, the relationship between adolescence and reward sensitivity is complex (Walker et al., 2017). Some studies find that adolescents overemphasize negative outcomes during learning (Rosenbaum et al., 2022), while others suggest that adolescent reward responsivity reflects changes in action initiation biases (the tendency to impulsively initiate actions) rather than reward learning alone (Pauli et al., 2023). These discrepancies highlight the complexity of adolescence, when pubertal hormones, motivational drives, and neural reward systems show reciprocal fluctuations (Braams et al., 2015, Walker et al., 2017). Results may differ based on the specific construct studied, such as reward responsivity versus action initiation (Pauli et al., 2023), novelty seeking versus uncertainty aversion (Nussenbaum et al., 2023), or risk versus ambiguity tolerance (Tymula et al., 2012). How development is measured (e.g., biological markers like testosterone vs. self-reported pubertal development) can also impact findings, with some studies finding that biological markers reveal stronger developmental effects (Barendse et al., 2024, Braams et al., 2015).

The sex differences that emerge with puberty further complicate puberty-related effects on brain and behavior (Beck et al., 2023, Holm et al., 2023). Boys typically exhibit higher reward sensitivity than girls in adolescence, corresponding with structural and functional differences in mesolimbic circuits (Barendse et al., 2024, Urošević et al., 2014, Walker et al., 2017). These differences have clinical relevance, as higher reward sensitivity is linked to better treatment outcomes for mental health disorders like anxiety (Norris et al., 2021, Sequeira et al., 2021), which become increasingly prevalent in girls during puberty (Khanal et al., 2022). Further research examining how individual differences impact reward sensitivity, and how reward sensitivity in turn impacts mental health trajectories, will be important for optimizing prevention and intervention strategies during the critical adolescent window.

#### 2.3.2 Exploration and risk-taking

Adolescence is characterized by heightened exploration and a greater propensity for risk-taking—defined here as making decisions with uncertain outcomes (Romer et al., 2017). These behaviors are thought to stimulate neuroplasticity and learning by offering opportunities for the brain to refine its circuits based on environmental feedback (Parr et al., 2024). Compared to adults, adolescents are more likely to engage in risk-taking, particularly when the outcome probabilities of each risk are ambiguous rather than clearly stated (Tymula et al., 2012, van den Bos and Hertwig, 2017). This readiness to embrace uncertainty is conducive to learning, as it allows adolescents to gather novel information that can guide future decisions, regardless of whether the immediate outcome is positive or negative.

The Exploration-Selection-Refinement (ESR) model provides a useful framework for understanding the role of exploration in learning and plasticity (Lindenberger and Lövdén, 2019). According to this model, learning occurs in three phases of plastic changes: Exploration, characterized by behavioral variability and risk-taking, when the brain probes available neural circuits to identify those capable of executing a task; Selection, where the best-performing circuits are prioritized; and Refinement, where selected circuits undergo further structural changes to stabilize processing, while unselected circuits are pruned. In line with the Exploration phase, adolescents exhibit a unique reduction in Pavlovian biases (automatic responses that typically guide behavior), leading to more flexible and less constrained decision-making compared to both children and adults (Raab and Hartley, 2020).

This exploratory behavior appears particularly adaptive for learning during the adolescent period, when individuals must navigate new and diverse environmental contexts. For instance, research suggests that adolescents engage in greater exploration and exhibit higher learning rates than adults in uncertain situations (Jepma et al., 2020), highlighting how exploration facilitates learning from novel environments. Similarly, the Life-Span Wisdom Model emphasizes the adaptive role of adolescent sensation seeking, exploration, and risk-taking, emphasizing how these behaviors foster improved decision-making and cognitive control as adolescents accumulate life experience and wisdom over time (Romer et al., 2017).

As individuals gain experience, their neural systems begin to specialize, and neurocomputational mechanisms guide the transition from exploratory to exploitative strategies that involve capitalizing on known resources and established neural circuits (Giron et al., 2023, Lloyd et al., 2023). While this stabilization is adaptive for adult life—when individuals are expected to engage with their environments independently and appropriately—it can also limit adaptability to new environmental challenges (Lin et al., 2020, Templeton et al., 2012). Recurrent neural network models illustrate this trade-off between flexibility and specialization; while synaptic pruning during learning enhances performance on familiar tasks, it simultaneously reduces the brain’s capacity to learn new tasks, reflecting the reduced plasticity of adulthood (Averbeck, 2022). This helps explain why adults, despite having more efficient neural circuits, often struggle to acquire new skills (e.g., mastering a foreign language).

In sum, adolescents’ increased exploration and risk-taking play an essential developmental role, fostering a broad capacity for environmental adaptation and the acquisition of diverse skills in preparation for adult independence (Ciranka and van den Bos, 2021, Giron et al., 2023, Lin et al., 2020, Parr et al., 2024, Romer et al., 2017).

#### 2.3.3 Model-free versus model-based learning

Reinforcement learning models shed light on developmental shifts in learning strategies, showing a progression from model-free (habitual, automatic responses) to model-based (goal-directed, deliberate) strategies as individuals transition from childhood to adulthood (Decker et al., 2016). Model-free learning relies on habitual behaviors formed through repeated action-reward pairings, without considering broader implications or long-term goals. In the context of the ESR model, model-free learning can be likened to the Exploration phase, where individuals engage in trial-and-error behaviors, often prioritizing immediate rewards over long-term considerations. Adolescents tend to rely more heavily on model-free strategies compared to adults, exhibiting greater exploration and reward-seeking behavior (Palminteri et al., 2016).

As adolescents mature, they begin to shift toward model-based learning, a more sophisticated strategy that incorporates a cognitive model of action-outcome relationships. This includes features such as counterfactual learning (learning from feedback about the unchosen option) and value contextualization (e.g., learning from both rewards and punishments; Palminteri et al., 2016). This transition can be mapped onto the Selection and Refinement phases of the ESR model, where individuals begin to develop goal-directed decision-making by anticipating the consequences of their actions and fine-tuning their neural and behavioral responses. While younger children predominantly use model-free strategies, model-based learning emerges during adolescence and strengthens into adulthood, supported by the maturation of prefrontal cortical and subcortical circuits that facilitate flexible and adaptive behavior (Decker et al., 2016).

As adolescents accumulate experiences and refine their learning strategies, they become better equipped to balance immediate rewards with long-term goals, aligning their decision-making processes with adaptive outcomes (Giron et al., 2023). Notably, improvements in fluid reasoning—the capacity to flexibly integrate independent pieces of information to arrive at a solution—have been shown to mediate the developmental increase in model-based learning (Potter et al., 2017). This cognitive shift is likely important for addressing the complex social, academic, and personal challenges faced during adolescence.

Integrating reinforcement learning models with the ESR framework can provide a more comprehensive understanding of how developmental and experience-dependent plasticity help facilitate the shift from exploration-driven, model-free learning to the more refined, goal-directed decision-making of model-based learning. Through initial increases in exploration and behavioral variability, adolescents can maximize learning from their new environments, refine their neural circuits, and develop adaptive, goal-directed decision-making strategies that support long-term success as they transition into adulthood (Giron et al., 2023).

### 2.4 Leveraging developmental science to optimize experiential learning

Development and learning are dynamic, interdependent processes that shape brain function and behavior throughout life. Both processes involve the acquisition and refinement of skills, guided by environmental stimuli, experiences, and social interactions. Whether acquiring language, mastering motor skills, or navigating social relationships, these processes rely on the brain’s remarkable ability to adapt and reorganize in response to external inputs.

Scholars have posited that development represents a specialized form of learning with distinct temporal constraints (Galván, 2010, Nelson, 2017). While learning can occur in virtually any neural circuit at any time, development is constrained by factors such as functional domain (e.g., somatosensory versus association cortices) and time window (e.g., pre- versus post-puberty). Further, development encompasses normative maturation-related processes (e.g., activational effects of gonadal hormones on social brain regions) and responses to environmental cues (Galván, 2010), shaping how the brain processes and prioritizes input, with long-term implications for behavior and learning (De Lorme et al., 2013).

Sensitive periods in development offer unique opportunities for experiential learning, making them promising windows for targeted intervention. A study probing the effects of learning and development on longitudinal brain network reorganization from childhood to adulthood found distinct and interactive effects of both factors (McCormick et al., 2021). Task experience increased learning rates and brain network modularity (i.e., the organization of the brain into specialized functional units), while age exerted nonlinear effects, with learning rates and network modularity increasing from childhood through adolescence and peaking in young adulthood. Crucially, the strongest impact of task experience on network modularity and learning rates occurred in young adulthood, when network modularity reached its developmental peak (McCormick et al., 2021). These findings suggest that experiential learning is most effective when aligned with the brain’s developmental trajectory, emphasizing the need for interventions tailored to specific developmental phases.

Social context plays a pivotal role in shaping adolescent learning by engaging the heightened social motivations characteristic of this stage, stimulating social plasticity (Ciranka and van den Bos, 2021, Cousijn et al., 2018, Nelson and Guyer, 2011). Studies have shown that adolescents are more likely to engage in exploration, exhibit prosocial behavior, and learn more efficiently in the presence of a peer than when alone (Lorenz and Kray, 2022, Silva et al., 2016, Sullivan et al., 2022), though the magnitude of these effects can vary based on individual differences (Lorenz and Kray, 2022). Adolescents’ willingness to entertain novel hypotheses in social domains suggests that social contexts enhance their capacity for flexible learning (Gopnik et al., 2017). While peer presence initially promotes exploratory behavior, adolescents rapidly adjust their behavior based on social feedback, reducing behavioral variability and risk for negative outcomes (Ciranka and van den Bos, 2021). This highlights how social plasticity facilitates transitions between the Exploration, Selection, and Refinement phases of learning, enabling adolescents to fine-tune their behavior and neural circuitry based on social feedback.

To maximize their developmental impact, programs geared towards adolescents may strategically combine elements like rewards and social interactions, while providing environments that are conducive to exploration and experimentation. Adolescents may be particularly responsive when social and emotional elements are combined, as research has shown that peer presence significantly influences decision-making in emotionally charged (“hot”) contexts, but had a lesser impact on decision-making in more deliberative (“cold”) contexts (Somerville et al., 2019).

Research into the neurobiological mechanisms of experiential learning in adolescence can provide valuable insights for designing effective interventions, identifying ideal participants, and determining optimal timing for implementation (Laube et al., 2020). The following sections will delve into recent findings that deepen our understanding of adolescent neuroplasticity and experiential learning, underscoring the profound significance of neural and social connections during this pivotal stage of life.

## 3 Environmental influences on adolescent learning and plasticity

### 3.1 Social relationships

Adolescence represents a pivotal period of social development wherein youth must learn to navigate diverse social landscapes and adjust their behavior to adhere to complex, context-dependent social norms (Crone and Dahl, 2012, Fuligni, 2019). As they progress through puberty, adolescents encounter increasingly complex social environments, requiring them to integrate inputs from multiple sources to meet evolving contexts and expectations. Heightened social plasticity during this time can enhance their ability to learn from social feedback, making social relationships particularly influential for guiding behavior (Blakemore and Mills, 2014, Spaans et al., 2020, Telzer et al., 2014). Recent research underscores the critical role of social relationships—especially those with parents and peers—in shaping adolescents’ neural and behavioral responses, thereby helping them develop and refine the skills needed to navigate their social world effectively.

#### 3.1.1 Parents: anchors in adolescent development

While peer relationships gain importance in adolescence, parental support remains crucial for supporting neural and behavioral functioning (Butterfield et al., 2021, Dandash et al., 2021). Supportive parenting can act as a protective factor against the negative impacts of poverty exposure on structural and functional brain connectivity, contributing to resilience among underserved youth. For example, in a sample of Black adolescents living in the rural South of the United States, more time living in poverty in adolescence was associated with reduced neural connectivity in brain networks supporting cognition and emotion regulation at age 25 (Brody et al., 2019). However, this association was not observed among individuals who experienced high levels of supportive parenting in adolescence, suggesting that support from parents was able to buffer the negative effects of poverty on brain connectivity in adulthood.

In another longitudinal study of youth ages 9–18 years, researchers identified a “high-risk” developmental trajectory, characterized by low white matter integrity and high anxiety symptoms in adolescence, and a “resilient” cluster, characterized by the same brain signature (low white matter integrity) but low anxiety symptoms (Buthmann et al., 2024). Adolescents living in disadvantaged neighborhoods were more likely to be in the high-risk cluster if they reported lower maternal warmth, while they were more likely to be in the resilient cluster if they experienced high maternal warmth. This suggests that supportive and warm parental influence may at least partially mitigate risk for altered brain development and mental health due to neighborhood socioeconomic disadvantage. Maternal warmth has also been shown to decrease risk for later anxiety and depression by reducing brain reactivity to criticism during adolescence (Butterfield et al., 2021).

For adolescents with early insecure attachment—meaning they showed difficulties forming a secure bond with primary caregivers in infancy and/or early childhood—parental presence can buffer against behavioral and neural dysregulation in response to socially aversive cues (Rogers et al., 2022). This suggests that parents can play a vital role in helping adolescents self-regulate and navigate challenging social experiences, especially for youth prone to social difficulties. Moreover, parental presence can promote adaptive decision-making in adolescence by modulating activity in socio-affective brain systems during fear conditioning and risk-taking (Abramson et al., 2024, Guassi Moreira and Telzer, 2018).

Apart from parents, siblings also hold significance in adolescent social relationships, impacting both behavior and brain function (Rogers et al., 2022). Further exploration of adolescent sensitivity to non-parental family members will be important for effectively leveraging existing social relationships to support adolescent functioning.

#### 3.1.2 Peers: catalysts for social learning and neural adaptation

The social reorientation of adolescence is associated with increased motivation to seek out peer interactions, offering valuable opportunities for refining social skills, practicing emotion regulation, and shaping personal identity within dynamic social contexts. The heightened neuroplasticity of adolescence, combined with the saliency of peer influence, makes peer-induced social plasticity a powerful tool for guiding social learning and behavior (Cousijn et al., 2018, De Lorme et al., 2013).

Research shows that adolescents exhibit elevated neural activity in reward-related brain regions when observing peers receive rewards, reflecting a unique sensitivity to peer outcomes (Koele et al., 2023). The mere presence of peers can promote altruistic behavior, with adolescents responding more quickly and prosocially when evaluating outcomes for peers compared to themselves, highlighting the accelerated social learning that occurs in peer contexts (Sullivan et al., 2022). Further, adolescents are more likely to engage in prosocial behavior after receiving prosocial feedback from peers, and those in classrooms with more prosocial peers displayed increases in prosocial actions over time (Busching and Krahé, 2020, Van Hoorn et al., 2016). This underscores the powerful role of peer-induced social plasticity in enhancing adolescents’ capacity to learn from their peers, fostering prosocial behavior and contributing to their overall socio-emotional development.

Given how significant peer relationships are for adolescents, as well as their heightened receptivity to peers’ social cues, peer-mediated learning—especially in socio-emotional domains such as emotion regulation—holds significant potential during this developmental stage (Sahi et al., 2023). Peer-induced social plasticity may stimulate activational and organizational neural changes, enhancing adolescents’ capacity to acquire new skills and knowledge via collaborative learning and social motivation.

Recent research using EEG hyperscanning, which measures brain activity in two people simultaneously, has shed light on the neural mechanisms underlying adolescents’ cooperative problem-solving with peers (Yang et al., 2023). Compared to adults, adolescents showed greater interbrain synchronization with their peer, meaning adolescent pairs had more similar activation patterns while engaged in the task. Further, interbrain synchronization in the visual system was associated with better task performance. These results suggest that adolescents are more attuned to social interactions with peers at a neural level, aiding in their cooperative learning and task performance.

Engaging in shared experiences with peers can help adolescents harness their social plasticity and tap into their brain’s inherent adaptability. These collective social experiences not only scaffold the development of large-scale neural systems crucial for social cognition and emotion regulation, but also forge new connections with other neural systems, enriching adolescents’ socio-cognitive repertoire and empowering flexible problem solving.

For instance, while canonical self-control brain systems undergo extensive development in adolescence, many individuals successfully regulate their behavior without robust activation in these regions, suggesting that they rely on other neural resources to compensate. Research suggests that greater activation in social brain systems can improve response inhibition in adolescents with weaker responding of canonical self-control regions—especially if they have extensive real-world social networks (Tompson et al., 2020). This suggests that peers not only influence social behavior but also facilitate broader neural adaptations that can facilitate both social and non-social behaviors.

Moreover, a recent study revealed a compelling association between adolescents’ degree of social connectedness—measured via their self-reported sense of closeness and belonging to others—and the integrity of white matter tracts in the brain (Driver et al., 2023), adding to the evidence that real-world social connections contribute to the long-term organization of communication pathways in the adolescent brain.

Collectively, this research underscores the potential of adolescents’ social plasticity to stimulate learning and foster positive development during this crucial stage. Adolescents forge social connections across numerous areas of their lives, each of which can offer unique support and perspective. Rather than acting in isolation, diverse social relationships can complement one another—for example, receiving support from family members increases adolescents’ likelihood of supporting their friends, and vice versa (Armstrong-Carter and Telzer, 2021). Thus, incorporating social support from diverse sources may be especially promising for supporting the multifaceted nature of adolescent development.

### 3.2 School and neighborhood environments

Adolescents navigate a myriad of varied and diverse environments that are impacted by micro- (e.g., personal relationships) and macro- (e.g., neighborhood characteristics) level factors. Among these environments, schools and neighborhoods stand out for their pivotal roles in shaping neural systems and facilitating adaptability in adolescence, ultimately influencing independence in adulthood.

#### 3.2.1 Schools: nurturing connections and learning

The school environment is a dynamic ecosystem with profound influence on neural and cognitive growth—extending well beyond the academic domain. As multifaceted hubs of development, schools provide a rich and structured setting where adolescents can engage in activities that stimulate cognitive, emotional, and social learning. Interactions with peers and teachers, exposure to academic challenges, and participation in extracurricular activities all provide invaluable opportunities for exploration and problem-solving, contributing to the formation of new synaptic connections and laying the groundwork for the refinement of complex brain systems.

Recent research highlights the importance of high-quality educational experiences in shaping the developing brain. Using standardized test scores from school districts across the United States, researchers estimated educational opportunity (average score from a school or district relative to the national average) and explored its association with white matter development in adolescents (Roy et al., 2024). Adolescents in schools with higher educational opportunity showed accelerated white matter development in tracts that support academic skills such as reading, even when accounting for relevant socioeconomic factors such as family income. These results suggest that the school environment promotes academic success in part via the structural development of brain networks during adolescence.

Test scores are not the only important aspect of the school environment for brain development, as research suggests that experience-driven neuroplasticity can be supported through a variety of topics and programs. For example, a study showed that in-school music training begun in high school impacted adolescents’ subcortical sound processing and accelerated the maturation of cortical auditory responses, which had beneficial effects on literacy skills (Tierney et al., 2015). This suggests that in-school programs across an array of topics can be beneficial for engendering healthy brain development and learning.

Furthermore, positive aspects of the school environment such as supportive teacher-student relationships, feelings of safety, and opportunities to participate in activities have been linked to intrinsic functioning of large-scale brain networks in adolescence (Rakesh et al., 2023), suggesting that the connections forged within the school environment align with coupling of spontaneous fluctuations between brain regions when the brain is at rest. Moreover, these patterns of neural connectivity were associated with adolescents’ mental health symptoms, suggesting that positive school environments foster mental well-being via their effects on developing brain systems. The reciprocal relationship between school experiences and brain network functioning may explain why higher levels of school engagement in adolescence predict favorable outcomes (e.g., higher income) in adulthood (Symonds et al., 2023).

Teachers can facilitate neuroplasticity and learning by employing pedagogical strategies that encourage engaging with the material (e.g., generating a scientific hypothesis versus being taught the scientific hypothesis), incorporate peer interaction (e.g., explaining a concept to a peer), and facilitate active student participation, while also providing explicit instructions and expectations to help scaffold planning, time management, and organization (Armstrong, 2016, Brault Foisy et al., 2020, Jansen and Kiefer, 2020). By promoting dynamic interaction with the material and incorporating social motivation in a structured setting, these strategies can enhance neural plasticity, thus supporting adolescents’ capacity for learning and adaptation (Goldberg, 2022). Through collaborative learning activities, group projects, and classroom discussions, adolescents not only acquire academic knowledge but also develop important social skills such as communication, cooperation, and empathy.

Teachers also have the important role of cultivating a classroom atmosphere where diverse identities and contributions are recognized, affirmed, and valued (Causton and MacLeod, 2020). By creating a safe and inclusive space, teachers can empower adolescents to express themselves, make decisions, take risks, and form meaningful social relationships. These positive interactions within the classroom environment not only foster a sense of belonging but also support the formation and refinement of neural systems underpinning socio-emotional development (Goldberg, 2022, Hawkins, 2021).

In addition to tailoring educational programs to leverage adolescents’ neuroplasticity, research suggests that providing adolescents with information about their brain’s dynamic capacity for change can enhance their learning outcomes (Goldberg, 2022, Jansen and Kiefer, 2020). By fostering a growth mindset and empowering students to take an active role in their learning process, educators can cultivate greater engagement, persistence, and resilience in the face of challenges (Goldberg, 2022).

#### 3.2.2 Neighborhoods: flexibility, uncertainty, real-world testing

Neighborhood environments complement structured school environments by providing real-world contexts for adolescents’ learning and development. While schools offer organized educational experiences, neighborhoods expose adolescents to a broader range of social, cultural, and environmental influences that can shape brain plasticity in unique ways. Neighborhoods can also offer opportunities for informal learning, hands-on experiences, and community engagement that are not always available within the confines of the school setting.

Neighborhood characteristics—including socioeconomic status (SES), exposure to green space, and even the quality of the air one breathes—can affect numerous aspects of adolescent brain and behavioral development. Adolescents living in similar neighborhood conditions across the United States show similar patterns of cortical thickness in the brain, suggesting that neighborhood effects can transcend geographical location (Dahl et al., 2024).

A widely studied neighborhood feature is socioeconomic status, comprising factors such as mean income, education level, and occupation within a community. Adolescents growing up in economically disadvantaged neighborhoods often face barriers in access to resources and opportunities, which can interfere with cognitive development and academic achievement, impacting the development of underlying neural systems (Hackman et al., 2021). Research suggests that lower neighborhood SES is associated with altered white matter tract development in early adolescence (beyond the effects of family income; Kulla et al., 2024), as well as reduced brain volume in prefrontal and hippocampal regions (Taylor et al., 2020), while early adolescent exposure to neighborhood violence has been linked to adaptations in brain regions such as the amygdala and hippocampus (Saxbe et al., 2018).

In a cross-sectional study of youth ages 8–22 years, age-related increases in functional brain network segregation was blunted in individuals living in low SES neighborhoods (Tooley et al., 2020), suggesting that neighborhood SES can alter functional trajectories. A longitudinal study added nuance to this finding, revealing that neighborhood poverty during childhood predicted reduced network segregation in early adolescence (ages 9–14 years), but this effect diminished from ages 15–19 years, with adolescents who lived in low SES areas in childhood showing “catch-up” increases in network segregation with increasing age (Michael et al., 2023). Importantly, this suggests that while lower SES neighborhoods may impact normative functional brain development from childhood to adolescence, there is potential for mitigating these effects through programs aimed at increasing neighborhood resources for adolescents.

Given the nonlinear and regionally heterogenous nature of adolescent brain development, certain neighborhood features may be especially impactful at specific developmental stages. Recent research using spontaneous cortical dynamics to index developmental neuroplasticity in individuals ages 8–23 found that neighborhood socioeconomic factors (e.g., mean family income) showed the strongest associations with intrinsic prefrontal fluctuations in middle adolescence, with peaks occurring around age 15 (Sydnor et al., 2023). This aligned with their findings that markers of plasticity in association cortices peaked in mid-adolescence (Sydnor et al., 2023), suggesting that brain systems essential for complex cognitive skills are especially malleable and adaptive to neighborhood SES in adolescence.

The peak in prefrontal plasticity during mid-adolescence may reflect an experience-expectant process, where the brain prepares to integrate normative developmental experiences, while adolescents’ increasing independence and tendencies toward exploration and risk-taking create opportunities for experience-dependent plasticity, allowing them to learn and adapt more effectively to their surroundings. This combination of maturation-related and experience-dependent plasticity could help explain why environmental factors such as SES have an especially strong influence on brain function during this time. Overall, these findings highlight the opportunity for targeted interventions focused on environmental enrichment (e.g., increasing access to physical and educational resources) during this neurobiologically sensitive period.

In addition to (and often intertwined with) neighborhood SES, physical characteristics of the neighborhood can influence adolescent brain development. For example, access to green spaces has been linked to positive cognitive function and mental well-being among adolescents (Sprague et al., 2022). Conversely, exposure to air pollution, such as particulate matter and nitrogen dioxide, has been associated with altered brain development, potentially contributing to cognitive deficits and increased risk of neurodevelopmental disorders (Cotter et al., 2023). Greater exposure to pollution was associated with heightened activity and connectivity in neural emotion processing systems when regulating negative emotions, and these neural markers contributed to worsening depression over adolescence—especially in adolescents living in high-pollution neighborhoods (Uy et al., 2024). While less frequently studied, noise pollution can also negatively impact brain and behavioral functioning, contributing to stress and cognitive impairments (Hahad et al., 2022).

Crucially, environments at various scales can exert interactive influences on adolescent brain development, presenting potential opportunities to either mitigate or amplify each other’s effects. Protective factors at one level (e.g., supportive teachers) can interact with other aspects of the environment (e.g., exposure to neighborhood violence) to impact adolescent brain and behavioral functioning. For example, positive parenting, family income-to-needs ratio, and school environments can moderate effects of neighborhood disadvantage on intrinsic functioning of large-scale brain networks (Rakesh et al., 2021, Rakesh et al., 2021). The numerous environments that adolescents encounter can therefore be leveraged to have downstream effects on other aspects of functioning.

Social aspects of the neighborhood environment such as collective efficacy, defined as the shared perception among residents of their ability to work together effectively to address common problems, maintain social order, and promote well-being, can potentially buffer other neighborhood adversities by providing social support at the community level. For example, approximately 1 in 4 adolescents living in large United States cities lived with half a mile of a past-year firearm homicide between 2014 and 17; however, exposure risk decreased as household income and neighborhood collective efficacy increased, and neighborhood collective efficacy buffered risk of firearm homicide exposure in adolescents with lower family income (Aubel et al., 2023). Strategies for helping communities build and utilize social ties, alongside income support, could help in reducing adolescents’ exposure to violence and other adverse experiences.

Rodent studies suggest that environmental enrichment in adolescence (e.g., by providing large cages with toys, climbing platforms, tunnels, wheels) can enhance synaptic plasticity in the hippocampus and protect against negative effects of chronic stress on the developing brain (Dandi et al., 2023, Murack et al., 2023). The specific aspects of the environment to enrich likely depend on developmental timing and the neural system being targeted; for instance, social enrichment has been shown to reverse the negative effects of adolescent social isolation in the rat hippocampus (Biggio et al., 2019), but it failed to reverse the effects of isolation in the mouse PFC (Makinodan et al., 2012), suggesting that different neural systems may have distinct sensitive periods during development. Since the PFC shows the greatest alignment with the socioeconomic environment in mid-adolescence (Sydnor et al., 2023), interventions focused on neighborhood enrichment—such as increasing access to physical and social resources—may be particularly beneficial during this period. However, further research integrating both animal and human studies is needed to better identify sensitive periods and design effective environmental enrichment programs tailored to adolescent development.

Additionally, the importance of macro-level programs and policies for supporting adolescent brain health cannot be overstated. For example, in the United States, offering more generous benefits to low-income families in states with high living costs can mitigate the adverse effects of poverty on brain structure and mental health issues, emphasizing the importance of macro-level interventions for underserved adolescents (Weissman et al., 2023). Moving forward, intervention strategies that implement change across both individual and systemic levels will be crucial for effectively supporting adolescents and fostering healthy brain development.

While significant progress has been made in understanding the influence of neighborhoods on adolescent brain development, many aspects remain understudied. For example, neighborhood features such as availability of transportation infrastructure, noise pollution, cultural programs and facilities, gentrification, and other environmental hazards (e.g., water contamination) may have distinct impacts on adolescent development. Further, societal changes frequently introduce new areas ripe for study—for example, the percentage of self-driving cars on the road may be an interesting topic of study in the next decades. Comprehensive research efforts should aim to fully grasp the multifaceted influences of the neighborhood environment on adolescent development.

Exploring how various neighborhood features shape adolescent development highlights the potential for interventions to target multiple environmental levels simultaneously (Silk et al., 2007). While neighborhood disadvantages (e.g., low neighborhood SES) can increase risk for negative outcomes, these effects are not set in stone. Instead, positive factors like parental warmth, peer interactions, supportive schools, access to green space, and neighborhood social cohesion can counteract neighborhood disadvantages by creating a strong support network for adolescents, enhancing their overall resilience and well-being. Interventions that implement changes across different layers of environmental influence, from family dynamics to community infrastructure, will be crucial for providing robust support to adolescents and promoting their positive development—even in the face of environmental challenges.

### 3.3 The role of experience in shaping developmental plasticity

Adolescence is a critical period marked by heightened neuroplasticity, making it a prime window for experience-driven brain development (Galván, 2010, Larsen and Luna, 2018). However, theoretical work by Frankenhuis and colleagues suggests that environments not only influence how plasticity is utilized but can also dynamically regulate the degree and duration of plasticity itself through meta-plasticity processes (Frankenhuis and Walasek, 2020). This meta-plasticity refers to the environment’s ability to alter how responsive the brain is to future experiences—particularly by modulating the thresholds for when learning can take place.

For instance, Frankenhuis’s work demonstrates that individuals in adverse environments may exhibit heightened plasticity as an adaptive response, enabling them to rapidly learn and adjust to unpredictable and challenging conditions. Conversely, enriched environments may extend the period of plasticity, allowing for prolonged and more nuanced learning opportunities (Frankenhuis and de Weerth, 2013, Frankenhuis and Walasek, 2020). This theoretical framework underscores the importance of not only providing enriching experiences during adolescence but also recognizing how different environments can modulate the brain’s capacity for change, either amplifying or constraining learning during sensitive windows.

Incorporating this framework into existing research on adolescent development suggests that interventions should be tailored not only to the timing of sensitive periods but also to the quality and type of environment adolescents experience. Interventions in enriched environments might focus on extending windows of opportunity for learning, while those in adverse environments may need to support more rapid, adaptive forms of learning that are contextually appropriate. This more nuanced understanding of how environments shape plasticity—both in terms of when and how it occurs—reinforces the need for targeted, context-specific strategies to support adolescent development.

## 4 Going forward: characterizing environmental influences in developmental neuroscience

### 4.1 The importance of large-scale longitudinal studies

To advance understanding of environmental influences on adolescent brain development, comprehensive and expansive investigations that follow individuals over time are imperative (van Atteveldt et al., 2021). Large-scale longitudinal studies such as the Adolescent Brain Cognitive Development^SM^ (ABCD) Study (https://abcdstudy.org/), Human Connectome Project (HCP; https://www.humanconnectome.org/), UK Biobank (https://www.ukbiobank.ac.uk/), and Autism Brain Imaging Data Exchange (ABIDE; https://fcon_1000.projects.nitrc.org/indi/abide/) are promising resources for unraveling the complexities of typical and atypical brain development and its interactions with the environment. These studies provide rich datasets that offer a comprehensive approach to investigating developmental trajectories by capturing diverse phenotypic, environmental, and genetic factors over time (Saragosa-Harris et al., 2022).

The ABCD Study® represents one of the most extensive longitudinal studies of adolescent brain development to date. Encompassing 11,880 adolescents recruited from 21 sites across the United States, ABCD measures a broad spectrum of neurobiological, environmental, and behavioral factors that can influence brain development and mental health outcomes. This is achieved through the integration of various neuroimaging techniques (e.g., MRI, fMRI), behavioral assessments, genetic analyses, and environmental measures. In fact, numerous studies discussed in this review drew from the ABCD dataset, underscoring its already substantial impact in propelling scientific understanding in the field.

In addition to the ABCD Study®, other notable large-scale neuroimaging initiatives have added to understanding of brain structure, function, and development across different populations and life stages. HCP focuses on mapping neural connections in the human brain, offering insights into brain connectivity patterns across various demographic groups and lifespan stages. It encompasses studies on young adults, lifespan trajectories, and disease-related investigations, including anxiety, depression, and Alzheimer's disease. Similarly, the UK Biobank provides a vast repository of health and genetic data from over 500,000 participants in the United Kingdom, including extensive brain imaging data alongside other health-related information. ABIDE aggregates functional and structural brain imaging data collected from laboratories worldwide to expedite understanding of the neural bases of autism spectrum disorders across development.

Incorporating data from these diverse large-scale initiatives enriches our understanding of neurodevelopmental processes, offering insights into the complex interplay between genetic predispositions, environmental factors, and brain development across different populations and contexts. Collectively, these initiatives—and countless others—contribute to the advancement of our knowledge and pave the way for targeted interventions and treatments to improve outcomes for individuals with neurodevelopmental disorders.

### 4.2 Adolescent neural urbanome

Accurately measuring the physical environment is key to understanding how specific environmental factors influence brain development and mental health outcomes, particularly during the environmentally-sensitive period of adolescence (Kühn and Gallinat, 2024, Sydnor et al., 2023). Recent work within the ABCD Study® has introduced the “Adolescent Neural Urbanome,” a comprehensive framework designed to enhance the characterization of environmental effects on neurodevelopment in large-scale studies of adolescents (Cardenas-Iniguez et al., 2024).

The Adolescent Neural Urbanome framework integrates geospatial data—such as neighborhood composition, socioeconomic indicators, and levels of urbanicity—with neuroimaging and behavioral assessments, enabling researchers to map the impact of urban living on developmental trajectories while minimizing participant burden. This framework captures a wide range of environmental subdomains, including neighborhood composition, exposure to pollutants, availability of amenities and services, access to natural spaces, air pollution, weather patterns, community health burdens, residential segregation, and legal biases.

By adopting this framework, researchers can gain a better understanding of how specific environmental features—whether enriching or adverse—interact with critical windows of neuroplasticity to influence learning and development during adolescence (Frankenhuis and de Weerth, 2013). This approach offers a valuable tool for identifying patterns that contribute to health disparities, informing public policy, and designing environments that foster positive developmental outcomes. As environmental neuroscience continues to evolve, frameworks like the Adolescent Neural Urbanome will likely be essential for shaping evidence-based strategies to promote healthy neurodevelopment across diverse urban settings (Cardenas-Iniguez et al., 2024, Kühn and Gallinat, 2024).

### 4.3 Challenges and complementary approaches

Despite their strengths, large-scale studies like ABCD have limitations that warrant consideration. One significant challenge is the measurement of puberty. Since ABCD begins tracking participants at ages 9–10, it may miss the early stages of puberty, especially adrenal and early gonadal processes. This is especially relevant for girls and certain ethnic groups, such as Black and Hispanic girls, who tend to enter puberty earlier than their peers (Cheng et al., 2021). Salivary hormonal data are collected only once per visit, limiting the ability to capture diurnal, monthly, or momentary fluctuations in hormone levels. Moreover, the annual assessment schedule may miss important developmental fluctuations that occur on shorter, sub-annual timescales.

While the ABCD Study® offers valuable population-level insights, its large sample size can also present challenges, such as identifying statistically significant findings that have limited practical relevance. Additionally, although the study aims to provide a representative sample of United States adolescents, the degree of representativeness may vary across outcome measures. Careful monitoring of the data relative to population demographics is necessary to ensure generalizability to the broader population.

To address these challenges, integrating findings from smaller, targeted studies with larger datasets like ABCD will be important for enhancing overall understanding of adolescent development (Tibon et al., 2022, van Atteveldt et al., 2021). Small-scale studies, which often employ more frequent and nuanced measurements—such as weekly hormone assessments or repeated neuroimaging sessions—can capture time-sensitive data that large studies may overlook. These smaller studies can focus on specific physiological or psychological processes, refining hypotheses and helping to interpret findings from larger datasets with greater precision, ensuring conclusions are both statistically robust and biologically meaningful. Additionally, small studies may be better suited to examining unique subpopulations, such as early developers or minority groups, whose developmental trajectories may not be fully captured in large, diverse samples. By combining insights from small and large-scale studies, researchers can form a more comprehensive understanding of adolescent development, ensuring that hypotheses are carefully formulated and findings are interpreted with appropriate caution to avoid misinterpretation.

### 4.4 Individualized and interdisciplinary science

Adolescent development is shaped by a complex interplay of neurobiological, social, and environmental factors, resulting in substantial heterogeneity in brain and behavioral outcomes across individuals. While group-level studies can uncover broad developmental trends, individual trajectories are influenced by diverse factors such as mental health symptoms (Bashford-Largo et al., 2021), sex differences (Romeo et al., 2016), socioeconomic resources (Rakesh et al., 2021), and lived experiences (Rudolph et al., 2021). Individual variability in environmental responsivity may significantly alter how plasticity unfolds during adolescence, leading to distinct developmental pathways that can either promote resilience or increase vulnerability depending on the environmental context (Frankenhuis and de Weerth, 2013). As adolescents explore their emerging identities within broader societal and cultural contexts, individual variability becomes increasingly significant, impacting neurobiological systems and behavioral and clinical outcomes (Mattoni et al., 2023, Mirpuri et al., 2019).

Recognizing the heterogeneity of adolescent experiences, scholars have emphasized the need for personalized approaches to studying development in this window. Precision brain mapping, for example, has gained traction as a method that captures detailed, reliable, individual-specific functional brain properties through extensive neuroimaging data collection from each participant (Keller et al., 2023, Labonte et al., 2024). This approach enables more accurate predictions of developmental pathways, offering insights that can inform targeted interventions. Such personalization is especially relevant for addressing common adolescent mental health disorders like anxiety and depression, where individual differences in adolescents’ environmental responsivity can significantly affect treatment outcomes (Norris et al., 2021, Sequeira et al., 2021, Zhou et al., 2024).

Interdisciplinary collaboration is equally essential for capturing the complexity of adolescent development across systems and timescales (Suleiman and Dahl, 2017). By integrating fields such as neuroscience, psychology, sociology, genetics, education, and epidemiology, researchers can explore the intricate mechanisms that drive adolescent development across a wide range of methodologies. Importantly, cross-species approaches that integrate animal and human studies can be particularly valuable for formulating and testing causal hypotheses across models and scales—especially in the field of developmental psychopathology (Meyer et al., 2023, Meyer and Lee, 2019). For instance, animal models can directly test the mechanistic effects of pubertal hormones on neurodevelopment and behavior, providing a foundation that human studies can build upon to better understand how these processes contribute to the subjective experience of mental health symptoms.

Recent methodological advancements have further expanded our ability to study adolescent development with precision and detail. Wearable technologies, for instance, enable real-time collection of data on adolescents’ behavior and environmental exposures (Bagot et al., 2018, Zhou et al., 2024), offering insights into how everyday environmental factors influence development. Neuroimaging techniques have linked individual and environmental factors to neural plasticity and connectivity dynamics (Keller et al., 2023, Sydnor et al., 2023), providing a more nuanced understanding of the maturational and experience-dependent processes affecting brain function during this key developmental window.

Neurofeedback, a non-invasive neuroimaging technique that allows participants to adjust their neural activity using real-time feedback, shows potential for leveraging adolescent neuroplasticity (Kirlic et al., 2022, Zich et al., 2020). Although neurofeedback research in adolescents is in its early stages, studies highlight its feasibility and potential. For example, adolescent girls learned to increase the “temperature” of a thermometer representing their prefrontal-amygdala connectivity, leading to significant connectivity changes that correlated with improvements in emotion regulation (Zich et al., 2020). Another study showed that adolescents successfully regulated their neural activity during mindfulness tasks, leading to improvements in self-regulation and body-focused mindfulness that were maintained at a one-week follow-up (Kirlic et al., 2022). By targeting individualized brain dynamics and harnessing the heightened neuroplasticity of adolescence, neurofeedback has the potential to induce meaningful neural changes—particularly when combined with cognitive and behavioral interventions in adolescent treatment strategies (Kirlic et al., 2022, Meredith and Silvers, 2024).

By adopting a cross-disciplinary and personalized framework, researchers can develop more tailored interventions that account for the variability in adolescents’ environmental responsivity. As findings replicate across species and methods, clearer pathways for intervention and treatment will emerge, strengthening the evidence base for understanding adolescent development. Ultimately, this approach will improve our ability to support positive developmental outcomes for adolescents by considering the unique contexts and experiences that shape each adolescent’s growth.

### 4.5 Adolescent-specific recommendations for supporting learning and development

Adolescence is a uniquely sensitive period where biological, environmental, and social changes converge to shape brain development and behavior. This period is marked by heightened neuroplasticity in regions responsible for executive function, critical thinking, and complex social abilities, as well as a heightened sensitivity to rewards and social influence. The following recommendations aim to optimize learning and development in adolescents by aligning interventions with their specific developmental needs.

#### 4.5.1 Targeting sensitive windows of social learning

The pre/peri-pubertal period is a critical window for social learning during which social isolation can have long-lasting detrimental effects on prefrontal development and social behavior (Bicks et al., 2020, Makinodan et al., 2012). Research from both human and animal models highlights the protective role of social enrichment during this time, which can buffer against stress and enhance socio-emotional resilience (O’Connor et al., 2024, Wade et al., 2019). These effects are driven by plasticity and meta-plasticity processes that allow the brain to not only adapt but also fine-tune its ability to remain flexible in response to future challenges (Frankenhuis and Walasek, 2020). Structured programs such as peer-based learning, group activities, and collaborative decision-making in early adolescence can therefore play a pivotal role in sculpting social brain circuitry, promoting positive social interactions, and mitigating the long-term risks associated with social isolation.

#### 4.5.2 Harnessing peer influence for positive development

The pivotal role of peers in shaping adolescent behavior can be strategically leveraged to promote learning and development (Busching and Krahé, 2020, Ciranka and van den Bos, 2021, Sahi et al., 2023). Programs such as peer-led activities, mentorship initiatives, and group-based learning provide structured opportunities for positive peer interaction, reinforcing the neural pathways associated with prosocial behavior and empathy and bolstering mental and physical well-being in adolescents (Busching and Krahé, 2020, Crone et al., 2022, Fuligni, 2019, Sullivan et al., 2022). These programs not only foster immediate learning but can potentially extend the brain’s social plasticity, further amplifying the effects of positive peer influence over time. Shifting peer group norms toward healthy behaviors—such as by incorporating peers into substance abuse prevention and mental health awareness strategies—can amplify the positive impact of peer influence, protecting against negative outcomes and reinforcing positive social outcomes (Cascio et al., 2015, Cousijn et al., 2018).

#### 4.5.3 Maximizing the impact of environmental enrichment

Emerging research has identified mid-adolescence as a period of heightened neuroplasticity in association cortices—particularly within the PFC—rendering it highly sensitive to environmental influences such as socioeconomic conditions (Sydnor et al., 2023). This developmental window presents a prime opportunity for targeted environmental enrichment interventions that provide access to positive resources like extracurricular activities, safe social spaces, and mentorship programs. By offering structured opportunities for exploration and engagement, these programs can enhance cognitive and emotional development and allow for continued neurodevelopmental flexibility, which could be particularly helpful for youth exposed to adversity (Frankenhuis and de Weerth, 2013). Through experience-dependent plasticity and meta-plasticity processes, environmental enrichment programs have the potential to mitigate the effects of environmental stressors, fostering long-term neurocognitive resilience and promoting healthy development into adulthood (Frankenhuis and Walasek, 2020, Joushi et al., 2021).

#### 4.5.4 Optimizing cognitive and neural interventions

Adolescence is a critical period for the development of executive functions like decision-making, impulse control, and long-term planning, supported by ongoing maturation of dopamine systems and association cortices (Larsen et al., 2023, Larsen and Luna, 2018). Interventions aimed at enhancing these cognitive skills through problem-solving, goal-setting, and impulse control exercises may be especially impactful during this time, especially when combined with reward-based incentives and social contexts to enhance motivation and engage neural decision-making systems. Given adolescents’ heightened neuroplasticity and flexible learning, combining digital tools such as neurofeedback, mindfulness apps, and gamified learning platforms with traditional cognitive-behavioral techniques may help maximize experience-dependent plasticity and promote healthy neurocognitive development (Laube et al., 2020).

#### 4.5.5 Teaching stress management and emotion regulation

Adolescents experience heightened stress reactivity and emotional volatility driven by the interplay of hormonal, neurobiological, and socio-emotional changes that accompany puberty (Guyer et al., 2016, Kann and Romeo, 2022). Therefore, programs that teach stress management and emotion regulation strategies are crucial for equipping adolescents with tools to handle emotionally charged situations and stressful life events (Eadeh et al., 2021, Pedrini et al., 2022). Incorporating neurofeedback techniques into these programs may further amplify their effectiveness by enabling adolescents to actively monitor and modulate the plastic neural circuits involved in stress and emotional responses, helping build adaptive coping mechanisms and socio-emotional resilience (Kirlic et al., 2022, Zich et al., 2020).

#### 4.5.6 Building multi-level support systems

As adolescents face new and evolving environmental challenges, establishing multi-level support systems that encompass family, school, and community settings becomes increasingly important. Supportive relationships with both peers and adults are vital for protecting against mental and physical health risks (Brody et al., 2019, Butterfield et al., 2021, Güroğlu, 2021, Rasalingam et al., 2017), and support at one level (e.g., parental involvement) can create a ripple effect that enhances other facets of an adolescent’s life, including peer relationships and school engagement (Armstrong-Carter and Telzer, 2021, Silk et al., 2007). Programs that implement change across these varied environmental contexts can help fill gaps in care, ensuring adolescents receive adequate support. Recognizing the interconnected nature of adolescent development, multi-level interventions can foster robust support systems, equipping adolescents with the resources and tools they need to navigate, adapt to, and thrive in their evolving environments.

#### 4.5.7 Encouraging identity development and autonomy

Adolescence is a pivotal time for identity formation during which individuals explore who they are, what they value, and how they fit into their broader social environments (Branje et al., 2021). Programs that encourage self-exploration, independent decision-making, and personal expression can play a vital role in supporting positive identity development. Providing safe spaces for adolescents to authentically explore their identities, particularly in high school, can promote mental health and resilience by allowing adolescents to build a strong sense of self and purpose within their broader environments (Mirpuri et al., 2019, Umaña-Taylor, 2016, Umaña-Taylor, 2023).

In summary, adolescence presents a unique window for targeted interventions that address the biological, social, cognitive, and environmental dynamics of this developmental period. Programs tailored to specific adolescent phases, whether focused on social enrichment, peer influence, or cognitive development, can promote positive development and support smooth transitions from childhood to adolescence and adolescence to adulthood. Continued research and policy changes are essential to refine and implement these interventions effectively, ensuring they align with critical periods of adolescent growth.

## 5 Conclusions and future directions

In this review, we explored the intricate relationship between adolescent brain development, environmental influences, and learning processes, emphasizing the crucial role of puberty in shaping adolescents’ sensitivity and adaptability to their experiences. At the heart of this developmental journey lies the concept of neuroplasticity—the remarkable capacity of the brain to rewire itself in response to experience. Adolescence is a critical window where this plasticity intertwines with learning, forming the foundation for adaptive behaviors and cognitive growth necessary for adult independence.

Puberty-related hormonal changes ignite adolescents’ curiosity for risk-taking and hunger for exploration, heightening neuroplasticity and priming the brain for experiential learning. By prioritizing decisions with uncertain outcomes, adolescents maximize learning from their diverse environments. Developmental neuroplasticity coincides with social plasticity, wherein social contexts further accentuate adolescents’ neuroplasticity and learning processes. Pubertal hormones enhance neural responses to social cues, increasing motivation to seek out meaningful social interactions and emotional exploration, while neural networks underlying socio-emotional regulation and empathy are fine-tuned.

Harnessing the combined strengths of neuroplasticity, social motivation, and learning can benefit the development of interventions aimed at promoting healthy adolescent development. Tailoring educational strategies to adolescents’ neuroplasticity can amplify their capacity for adaptive learning and cognitive growth. Similarly, leveraging social contexts to foster meaningful interactions and emotional connections can enrich their learning experiences, fostering resilience and well-being. Programs that increase positive environmental influences (e.g., environmental enrichment) during adolescence hold promise for supporting healthy brain development, with adolescents’ intrinsic neuroplasticity increasing their potential to improve developmental outcomes and mitigate adverse environmental challenges during this period.

Moving forward, it will be imperative to continue unraveling the complexities of adolescent neuroplasticity, learning, and their interplay with environmental factors. Longitudinal studies and multidisciplinary approaches offer valuable insights into the mechanisms underlying this dynamic relationship, guiding the development of targeted interventions and policy initiatives. Moreover, frameworks such as the Adolescent Neural Urbanome offer innovative methods to use geospatial information combined with neuroimaging data to map regional policies and environmental factors to brain development, facilitating targeted interventions and policy decisions.

In conclusion, this review underscores the crucial impact of environmental factors in adolescent neuroplasticity and learning. Findings from recent studies emphasize how various contexts, including social, school, and neighborhood environments, shape the developing brain during puberty. Characterizing the multifaceted nature of adolescence requires comprehensive and interdisciplinary approaches that incorporate diverse contexts, varying timescales, cross-species methods, and intersectional perspectives. By further exploring the mechanisms underlying adolescent plasticity and learning, as well as advocating for targeted interventions and scientifically informed programs and policies, the field can better support adolescents in harnessing their unique abilities to navigate the complexities of their changing world on their journey to adulthood.

## CRediT authorship contribution statement

**Andrew J. Fuligni:** Writing – review & editing, Supervision, Conceptualization. **Adriana Galván:** Writing – review & editing, Supervision, Conceptualization. **Amanda E Baker:** Writing – review & editing, Writing – original draft, Conceptualization.

## Declaration of Competing Interest

The authors declare that they have no known competing financial interests or personal relationships that could have appeared to influence the work reported in this paper.
